# Models of Emergency Departments for Reducing Patient Waiting Times

**DOI:** 10.1371/journal.pone.0006127

**Published:** 2009-07-02

**Authors:** Marek Laskowski, Robert D. McLeod, Marcia R. Friesen, Blake W. Podaima, Attahiru S. Alfa

**Affiliations:** Internet Innovation Centre, Electrical and Computer Engineering, University of Manitoba, Winnipeg, Manitoba, Canada; University of Giessen Lung Center, Germany

## Abstract

In this paper, we apply both agent-based models and queuing models to investigate patient access and patient flow through emergency departments. The objective of this work is to gain insights into the comparative contributions and limitations of these complementary techniques, in their ability to contribute empirical input into healthcare policy and practice guidelines. The models were developed independently, with a view to compare their suitability to emergency department simulation. The current models implement relatively simple general scenarios, and rely on a combination of simulated and real data to simulate patient flow in a single emergency department or in multiple interacting emergency departments. In addition, several concepts from telecommunications engineering are translated into this modeling context. The framework of multiple-priority queue systems and the genetic programming paradigm of evolutionary machine learning are applied as a means of forecasting patient wait times and as a means of evolving healthcare policy, respectively. The models' utility lies in their ability to provide qualitative insights into the relative sensitivities and impacts of model input parameters, to illuminate scenarios worthy of more complex investigation, and to iteratively validate the models as they continue to be refined and extended. The paper discusses future efforts to refine, extend, and validate the models with more data and real data relative to physical (spatial–topographical) and social inputs (staffing, patient care models, etc.). Real data obtained through proximity location and tracking system technologies is one example discussed.

## Introduction

### Scope

Hospitals represent a promising area where modeling and simulation can be effective tools in evaluating patient access and patient care policies and efficiencies. In many cases, the operations of an emergency department (ED) are over taxed, as they represent the necessary compromises between competing priorities. Although policies and practices evolve over time and best efforts are made to reduce patient wait times and other patient care parameters, often there is little quantitative analysis or feedback in the process.

In this paper, we apply both agent-based model (ABM) and queuing model (QM) techniques to the operations of an ED, specifically with respect to patient access and patient flow through the ED. The objective of this work is to gain insights into the comparative contributions and limitations of each respective technique. The broader objective of the work is to contribute empirical input into healthcare policy and practice guidelines related to patient access and patient flow. Currently, our work has generated general models (ABM and QM) relative to patient access and patient flow in EDs. These are currently built on relatively simple models of the physical layouts and social processes within EDs. Although derived from input from healthcare experts, the models represent low-level, coarse-grained models of EDs, as these are a suitable starting point from which to evaluate the model's validity. These general models are presented in this paper, and they provide an opportunity (within and between the ABM and QM models) to investigate the relative sensitivities and impacts of various model parameters on patient access and patient care indicators.

In addition, several concepts from telecommunications engineering are translated into this modeling context. The framework of multiple-priority queue systems and the genetic programming paradigm of evolutionary machine learning are applied as a means of forecasting patient wait times and as a means of evolving healthcare policy, respectively.

In general, the ABM approach is applied in this work to investigate scenarios for resource optimization within the operations of an ED (for example, staffing scenarios). The QM approach facilitates quantitative analysis of operational parameters in EDs (for example, wait times). However, in both cases, the intent is to carry out predictive modeling with increasingly empirical inputs, which not only provide greater and more complex insights into the operations of EDs, but feed into the improvement of the ABMs and QMs themselves. To that end, this paper discusses the opportunities and future efforts to refine, extend, and validate the models with more data and real data (vs. simulated data). Real data obtained through proximity location and tracking system technologies is one example discussed. Future opportunities and efforts will also focus on refining, extending and validating models with a more complex range of physical (spatial–topographical) and social models (staffing, patient care models, etc.), such as those extracted from real time location systems and emergency department information systems, respectively. Augmenting the range of agent behaviours and interactions in an ABM is an interdisciplinary enterprise, and future efforts will rely heavily on input from healthcare experts.

### Background

Agent-based modeling is systems modeling, approached from the ground up or from the perspective of its constituent parts, in order to build an aggregate picture of the whole. Systems are modeled as a collection of agents, their individual behaviours, and their interactions. Agents are autonomous decision-making entities able to assess their situation, make decisions, and compete with one another on the basis of a set of rules. ABM's conceptual depth is derived from its ability to model emergent behaviour that may be counterintuitive or, at minimum, its ability to discern a complex behavioural whole that is greater than the sum of its parts. ABM provides a natural description of a system that can be calibrated and validated by representative expert agents, and is flexible enough to be tuned to high degrees of sensitivity in agent behaviours and interactions. ABMs are particularly well suited to system modeling in which agent behaviour is complex, non-linear, stochastic, and may exhibit memory or path-dependence [1].

A considerable focus of the applications of ABMs has been on community-level epidemic modeling in human populations (see, for example [2]), as this is an important public health and policy issue with far-ranging health and economic impacts. Within healthcare settings, a literature exists with respect to applying ABMs, alone or in complement to other techniques, to the operations of EDs. In general, this literature addresses system-level performance dynamics, quantified in terms of patient safety [3], economic indicators [3], [4], staff workload and scheduling [5], [6], and patient flows [7], [8]. While this literature addresses system-level operational concerns during periods of typical operation or stasis, there is also a literature on modeling of healthcare operations during critical incidents like disease outbreaks and terrorist attacks [9], [10], [11]. However, authors agree that relatively little work exists in applying ABMs to healthcare policy development [12]. Our own prior work includes both the development of a large scale (community-level) agent-based epidemic model [13], and more recently, an ABM for hospital acquired infections [14].

Complementary to ABMs, queuing-based modeling represents a well established and vetted methodology in operations research, with extensive applications in the service industries. Even though its application to healthcare is not new, this application has grown more recently, and the need is more recognized. For example, the forthcoming CORS/INFORMS 2009 conference in Toronto, Canada has devoted 15 sessions to healthcare applications, of which at least five are focussed on the applications of queuing theory. A recent issue of the flagship journals of INFORMS, Operations Research (Vol. 56(6)), was a special issue on Operations Research in Health Care. There is a clear recognition that QM can be applied creatively to understand and estimate the expected performance of the service processes in a healthcare system. Applications of QM in healthcare range from using it to study flows in EDs [15], analysis of delays for medical appointments [16], and for determining hospital bed requirements and allocations [17]. Most importantly, the model can be used to assess the operations of the healthcare system under different ‘design’ scenarios. By identifying the service points in a healthcare system, the associated topological linkages between these points and the stochastic processes that characterize the arrival process of patients and service processes of healthcare staff, one can apply a QM to quantitatively describe patient flow through the systems as well as waiting times in the system. QMs allow us to assess different configurations of service nodes and triage rules. Most of the existing models for healthcare are strictly queuing models.

In addition, technologies are emerging that can be leveraged by hospitals to improve patient care. Two of the more obvious technologies and applications include intra- hospital tracking and internetworking. These technologies can allow for a more distributed approach to managing a number of interacting EDs. This is incorporated into one of the ABM applications described in this paper, relative to evaluating ambulance redirection or other patient diversion policies. Previous work by the authors presented a specific emergency department data collection application and architecture and extended it to a wide area Hospital/ED/Ambulance and patient diversion framework [18].

## Results and Discussion

### Basic ABM Framework

**Figure 1:**
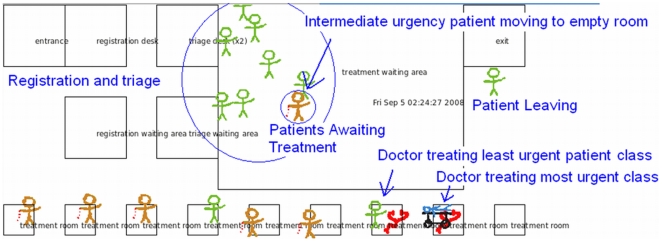
Screen Capture of the Basic ABM Simulator.

Our work has focussed on an object oriented (OO), open-source visual simulator which can be used to gather data from a patient flow monitor information, applied to analyzing and forecasting patient waiting times. The simulator was written using C++ and makes use of the Qt4 API for cross platform windowed applications [19]. By virtue of the open-source nature of both Qt4 and our code, the Beta stage of the project will benefit from other researchers' customizations and extensions. This would not be possible with an off-the-shelf proprietary solution rather than an open-source paradigm. Qt4 also allows us to deploy the simulator on Windows, Mac, or Linux. A screenshot of the simulator window is shown in Figure 1. The spatial aspect of the visualization reflects the spatial nature of the underlying data sources.

Further details of the ABM simulator are presented in [18]. The OO paradigm allows for instantiation of EDs and allows for communication between EDs. In an actual healthcare setting, patient information could be effectively conveyed on dashboards within the participating EDs, as well as on a dashboard at a more centralized control location. The flexibility and reuse of code facilitated by the OO architecture enables instantiation of multiple EDs by sub-classing or extending existing classes to allow communication between instances of EDs. Each ED maintains a collection of patient generators, agents representing patient care points (registration, triage, etc.), patient agents, and staff agents. A special controller agent is used to mediate patient flow through the ED process. Creating subclasses of the controller is necessary to handle variations on the basic ED processes, in order to reflect different policies for individual EDs being modeled. For example, one could create a subclass of the controller for an ED that allows for bedside registration for all patients, versus an ED that requires most patients to register at a desk (as per the current implementation). While the current model represents coarse-grained ED operations, the classes that represent staff and patient care points may also be subclassed to reflect procedures that vary between EDs. As the model is refined and extended, patients can also be subclassed.

At every simulated time step, all relevant agents statuses are refreshed. For example, in the general model, patients move between nursing, waiting, and treatment areas, as well as tracking time spent in each activity. Patient care points (e.g. nursing stations) count down the time required to process patients for the relevant activity. Patient generators model a Poisson arrival process for each patient class (i.e. classified by urgency of care required). At each time step, each decides whether to introduce a new patient.

In the current model, patient arrival rates and service times are based on estimates obtained by other researchers [20]. Further work will focus on driving the simulations on real world data collected in real time, such as that proposed in [21], where RFID or other proximity location system data would be used to augment the simulator. Further, the current model is able to place functional areas of the ED at arbitrary locations. Future work will integrate real spatial–topographical data taken from floor plans of the EDs wish to simulate. Floor plans from various EDs are readily available, such as the Halifax QE2 emergency department available at [22]. This type of topographical information is becoming more readily available and extremely useful for modeling purposes.

### Basic Analytic Queuing Model

A further aspect of the work is to implement queuing models (QMs) as a complementary technique to ABM, as a means of gaining complementary, comparative, and high level insights into afore-mentioned healthcare applications. In contrast to simulation, a QM is analytic, able to provide insight expediently but often in exchange for accuracy. The current application was to develop a general baseline model of an ED suitable for comparison with the ABM simulation. Similarly, multiple EDs can be modeled as a network of queues, augmented with numerical techniques and assumptions of dependencies. The work fits into the overall objective of combining the ABM with the analytic QM in a hybrid that would be both fast and accurate. As the work develops, it may become evident that one approach is preferable, more applicable, or more insightful than the other, depending on the results desired.

**Figure 2:**
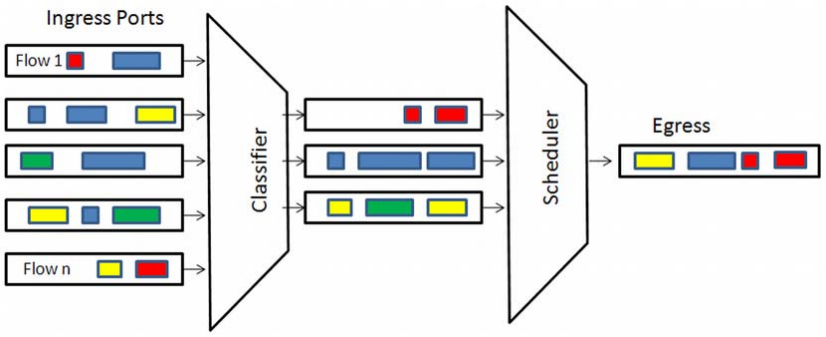
Queues within Telecom Equipment.

In applying QMs to healthcare applications, the telecom analogue remains very applicable, as there is both a vast literature on simulation as well as on QMs. In addition, networks are topographically somewhat similar to a network of EDs and similar to flows within an individual ED. The following example could represent part of the patient flow through an ED, framed within a QM: a patient would arrive with an injury, be registered, triaged, scheduled for diagnostic services and treatment, and discharged. Figure 2 illustrates an analogous and familiar queuing situation in a telecom context. A point to note is that although QMs have been used to improve and analyze a wide variety of processes, the telecom field is one with close correspondences to many healthcare scenarios. For example, there are queuing phenomena common in telecom networks that decrease system performance that would have a corresponding analogue in healthcare, such as head-of-the-line blocking. Extending this notion, an analysis of algorithms applied in telecom networks to optimize system performance may also have novel applications to healthcare. An example of head of the line blocking in a healthcare setting may be a patient ready for discharge from the ED, but waiting for an as-yet unavailable bed on a ward.

Complexities are added to the model in that the arrival rates of heterogeneous patients are not governed by well-behaved statistics. In addition, the queues may be pre-emptive, in that if a person arriving with a serious injury would pre-empt others waiting in various queues. Available ED resources, including physical resources (beds, equipment, etc.) and human resources (nursing staff, diagnostic staff, physician staff, etc.) further add complexity to the model when compared to a communication or data network. However, some of the basic and overall performance measures are similar. For instance, the total service time of a patient in the system (entry to exit) is a measure of interest in both a data network as well as an ED. In a network, analogous policies such as prioritization are used to prevent unbounded delay (time spend in the ED) from occurring for important traffic (more serious patients).

In terms of providing information to healthcare staff and administrators, a queuing figure provides an inherently familiar visual means of displaying bottlenecks in an ED. In general, patients in individual queues are often in a common waiting area. A dashboard display in the ED illustrating the various queues in real time could be a valuable means of displaying a snapshot of what is going on in terms of patient flows, routing, and delays.

**Figure 3:**
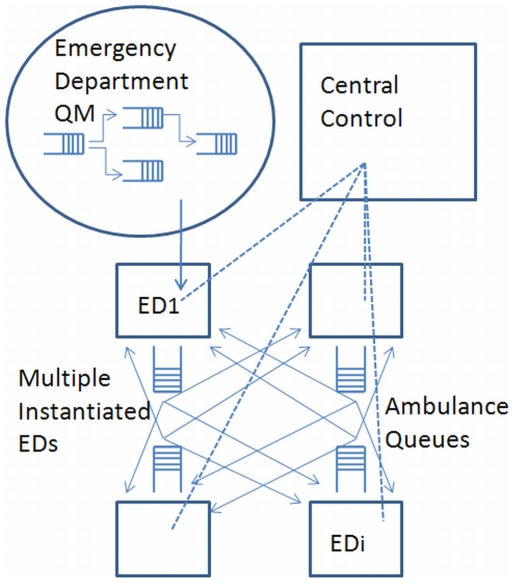
A Network of Emergency Departments Connected by Ambulance Queues.

As an extension to a single ED modeled within a QM framework, an inter-hospital network of EDs will more closely resemble a complete graph, as in practice any ED could redirect patients to any other ED (Figure 3). In reality, there would also be a hierarchy of EDs, as some may be regionally designated trauma centres and/or priority centres for specific types of presenting injuries. In addition, geography may make it more practical to divert patients to closer EDs, as more distant EDs may add to a patient's overall delay or time spent in the system. The complete graph of a network of EDs contrasts modern telecommunications networks in that communication networks are sparse graphs relaying packets of data as they traverse the network. While a modern telecommunication network does not closely resemble a network of EDs, the various *services* on a modern network do. However, a decided advantage of modeling multiple EDs as opposed to a telecom network is the feature of central control. Computer communication networks lack centralized control (although traditional telephony networks rely on control in establishing a path through a network on which an actual call can take place).

To address some of the complexities of networked inter-hospital QMs, a degree of simplification can be achieved by focusing the model on patient diversion for high priority patients. This approach would accurately model the high priority queues within EDs, with all non priority patients representing background noise in the system. Information required by the patient diversion scheduler in a coarse-grained approach to this scenario would include the patient triage level, the estimated delay at the initial receiving ED, and estimated transport delay and estimates of delay at the target ED. By necessity, the system would be a non-preemptive priority queue, in that, once a lower priority patient is in transit they would not be pre-empted in transit.

A variation on the above scenario which reduces the degree of simplification is to explicitly simulate the high priority patients and aggregate all other patient flow. As such, the explicit, high priority patients, as well as various policies and protocols would be modeled in detail. Modeling would not keep track of individual patients, but only their aggregate impact on delay of high priority patients at individual EDs. In this scenario, the aggregated (background) patient traffic would be context (ED) specific.

A final extension to this work is to consider a network of EDs with some degree of hierarchy, based on ED capacities, priorities, and capabilities. In this case, patient diversion would not only consider queue lengths at various EDs, but also the priority level of patients. One may see, for example, the diversion of a less critical patient to a hospital with fewer resources, rather than contributing to a queue of low priority patients at a regional trauma centre.

## Analysis

### ABMs for Patient Access to Emergency Departments

**Figure 4:**
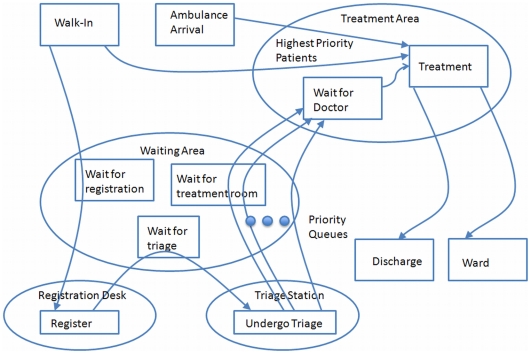
Model of Emergency Department Patient Service.

Initial efforts have focussed on the modeling basic aspects of an ED treatment process, as depicted in Figure 4. The current model is representative of a simple framework, suitable for simulations that provide insight into the relative sensitivities and impacts of the simulation parameters without necessarily quantifying them. As well, the current model can be validated on an ongoing basis, before and concurrent with adding requisite complexities.

In the current model, patients arrive either by ambulance or walk in. Patients in need of immediate care are sent directly to a treatment area. All ambulance arrivals, and a small fraction of walk-ins are considered to be in need of immediate care. Walk-ins that do not require immediate care proceed to the registration desk. If the registration desk is busy with an earlier arrival, the arriving patient waits in a queue. Once the registration process is complete, the patient proceeds to the triage station. If the triage station is busy, the patient again waits in a queue. Nursing staff at the triage station assign the patient a priority level based on the severity of their condition. The arriving patient then waits with other patients in what is effectively a priority queue to be assigned a treatment room.

This model is similar to many multiple priority queue system found in many telecommunication technologies, such as 802.11e [23]. 802.11e proposes wi-fi extensions for improving the more common 802.11 standard, through the use of priority queues serving various classes of traffic. When applied to healthcare, the utility of using analogues from telecom as a benchmark or reference is that these amendments are usually well vetted and studied and can be readily leveraged.

Once assigned a treatment room, the patient waits for physician staff to attend and provide treatment. It is assumed that treatment staff are physicians, and that they will treat the patients in order of urgency, then in order of arrival. Upon completion of the treatment, the patient leaves the system, and both the treatment room and the doctor become available for another patient.

Model parameters include (but are not limited to) the number and types of agents, the range of agent behaviours, the range of agent interactions, the spatial-topographical nature of the environment, and the nature of the processes being simulated. In building an ABM from the ground up, the initial simplicities are necessary to validate the model qualitatively and on an ongoing basis as it is refined and extended. By its very nature, further efforts in ABM development focus on expanding the range and nature of model parameters to better reflect real-life environments and social processes, and this applies to the range of work described in this paper.

### ABM Simulation of Staff Allocation

A series of simulations was carried out relative to staff allocation in an ED, to investigate the utility of the ABM framework for optimizing resources and making informed policy decisions. The first scenario we investigated, while simple, illustrates the effects of changing staffing levels, using multiple performance metrics.

**Figure 5:**
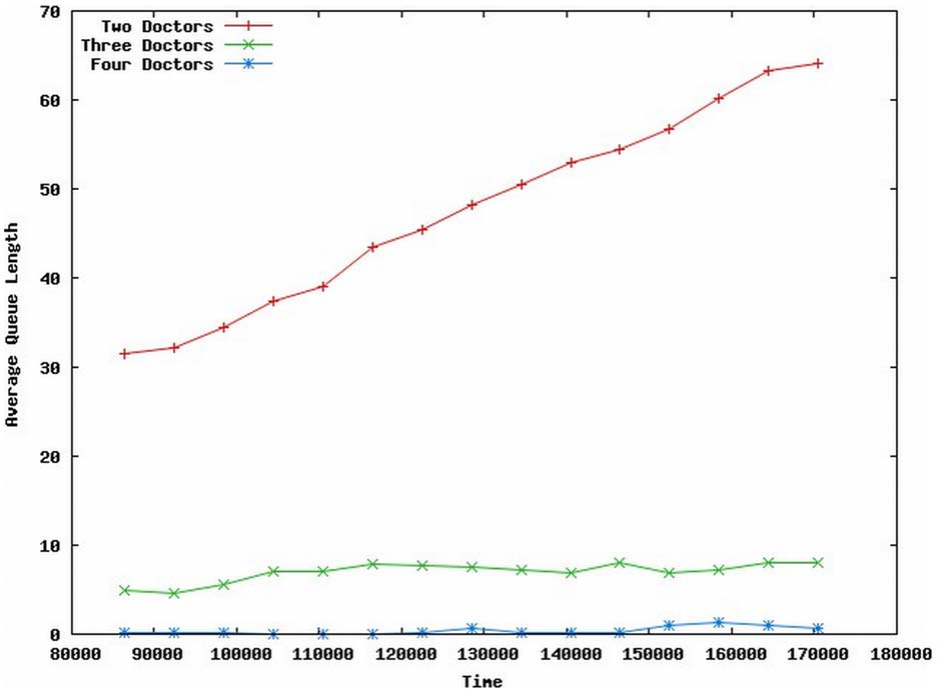
Average Queue Lengths for Varying Number of ED Doctors on Duty.

In this scenario, we simulated the basic ED scenario described earlier, with Triage Classes, Service Times, and Patient Arrival rates based on [20]. We compared three different staffing scenarios of two, three, and four doctors working in the ED. The simulation was allowed a “warm-up” period of 24 hours, then observations were made during the following 24 hours. Ten independent trials were run; average treatment queue length is shown in Figure 5. Alternatively, doctor utilization or individual patient waiting times can also be instrumented. The staffing simulation is qualitative, but represents one instance of this type of model that can be investigated at individual hospitals.

Intuitively, the simulation results appear reasonable. Figure 5 shows the average number of patients waiting for treatment as a function of time (in seconds). The value of the results at this stage of model development is qualitative: the ED model staffed with two doctors is understaffed, evidenced by a continually increasingly patient queue. At the other end of the continuum, the ED staffed with four doctors results in a patient queue of near-zero; however, corresponding results indicate that the doctors are underutilized. This allows discussions to occur relative to resource allocation and optimization, in this case, physician resources.

In refining the simulation, one would seek to apply context-specific patient, staff, and patient care parameters, as discussed earlier. Where individual EDs are instances of a regional hospital authority, further extensions of the work are to model multiple facilities and thereby provide a means of assessing patient diversion policy between facilities.

Prediction based on modeling and simulation is extremely difficult and potentially error prone. Confidence can be enhanced as the system is in operation and predictions tracked. Our conjecture is that the model of individual or interacting emergency departments, augmented with whatever available empirical data is available, would still be preferable over loop policy decision making. Sensitivity analysis associated with both the ABM engine (numerical stability) as well as validating the null hypothesis in term of a policy's effectiveness is still required.

In refining the simulation, one would seek context-specific patient arrival rates. For our purposes, the individual EDs are instances of a regional hospital authority; this facilitates the opportunity to model multiple facilities and thereby provide a means of assessing patient diversion policy between facilities.

### ABMs for Inter-hospital Patient Diversion

While the previous sections focussed on ABM simulation of patient access and patient care within EDs, this section provides an overview of how a modeling system could be extended between hospitals and integrated within a regional health authority informatics system. The discussion is model-agnostic, but for discussion purposes, an ABM is presented. An ABM of an ED is useful on its own [3], and we propose that its utility can be enhanced when combined with real data captured via tracking technologies and networking capabilities. In this specific application, the ABM is a distributed model across a number of regional hospitals, with an emphasis on utilizing data collected and analyzed in near real time. A novel aspect of the present model is the use of congestion avoidance algorithms from telecommunications engineering redeployed as a model for evaluating patient diversion policies. Again, the current models would benefit from the addition of real data in near-real time; such data is becoming increasingly available, although in some instances it may have to be inferred or estimated [21]. Subsequently, this data needs to be shared among regional hospital and health care facilities. Availability of and access to data are both technical and political challenges, although they are optimistically considered to be surmountable.

**Figure 6:**
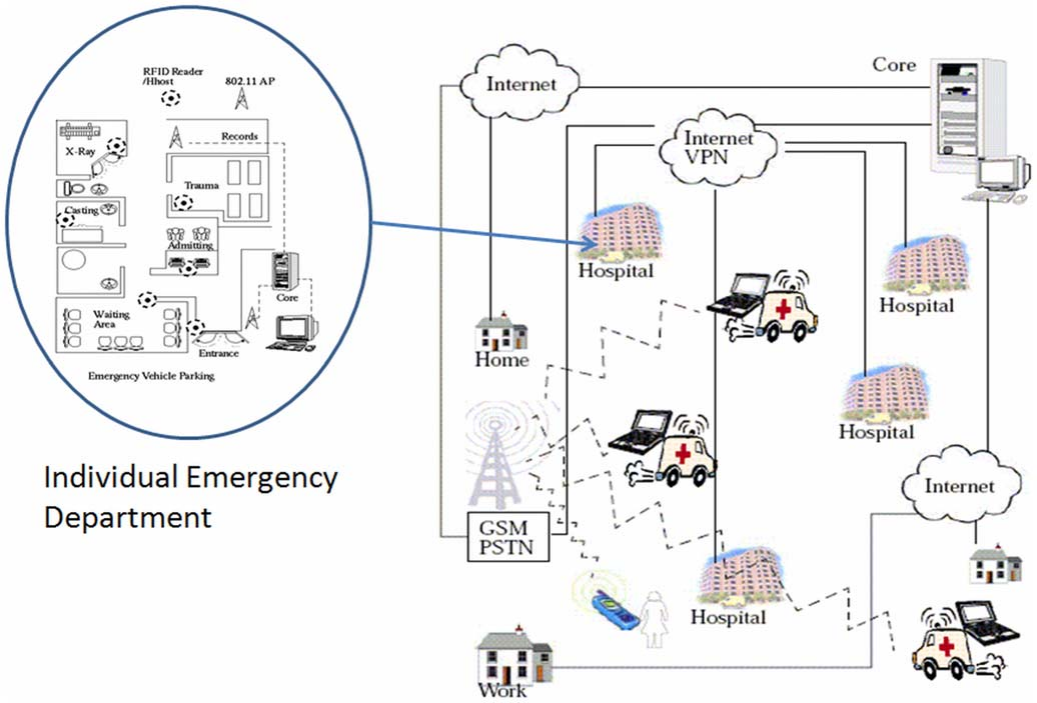
Wide Area Deployment of the Framework Illustrating the Major Stakeholders or Agents.

Figure 6 illustrates a wide area scenario incorporating participating hospitals and emergency service vehicles. In the wide area scenario, each hospital ED is equipped with tracking and queue monitoring and collection systems, where data would be in turn made available at a decision support center and would serve as inputs to the ABM support system.

At present, ambulance diversion is principally based on best effort reporting and good-faith operation based on regional guidelines, an example of which can be found at [24]. In other situations, diversions are posted, effectively preventing a patient from being brought to the posting facility. If these practices are done in an ad hoc or mutually exclusive fashion, they are unlikely to be optimal. In addition to these valuable heuristics, ED modeling can benefit from algorithms associated with conceptually similar areas such as the Internet and congestion avoidance schemes that deal with overcrowding of routers. In this application, we adapted the Random Early Detection (RED) algorithm [25] as a candidate for consideration when attempting to optimize ambulance or patient redirection, based on ED congestion information. This is an ideal initial algorithm for adaptation, as it has many of the attributes well suited to improving system throughput. RED accommodates limited bursts and can be effective, even when there is limited sharing of information between EDs. The RED patient diversion policy will serve as a baseline for comparison for machine learnt policy or optimizations discussed in a later section.

Furthermore, modeling extensions that add intelligence include the ability to notify and receive information from ambulances and other emergency vehicles. The actual communication services would most likely be over GSM or similar communication infrastructures where they exist. In the ABM, these services can be modeled as messages between agents, and the ABM platform would assist in optimizing ambulance diversion policies. Other considerations include estimates of emergency vehicle travel time, as these factors would be of significance in an effective model. Although beyond the current scope, the proliferation of GPS and mapping technologies allows these estimates to become empirical inputs to the multiple ED simulation. Future extensions will focus on data sources on vehicular congestion and congestion avoidance, as additional empirical refinements to the modeling efforts [26].

### ABM Simulation of Collaborating ED Data Infrastructure

In addition to simulations relative to staff allocation in an ED, the work further simulated a collaborating ED data infrastructure as described earlier. Real-time ED status data collected with RFID technologies would be disseminated and utilized in real time, informing ambulance and other emergency services, as well as individual citizens (perhaps through a web portal), of the near real-time status of EDs on a community-wide scale. This would allow patients and care providers to make more well-informed decisions on which ED to visit, based on current and projected wait-times. In order to forecast future patient wait times, the simulation can be run into the future a number of times, keeping track of the wait times experienced by patients arriving at future times – until some reasonable level of confidence is reached. During this process, the visualization can be disabled in order to speed multiple trials.

Since these types of systems are not yet in place, the well-vetted RED algorithm was used to model this process. As a method of network congestion management, senders of data over the network (typically the Internet) are implicitly notified of network congestion by having their data packets (discrete chunks of data) probabilistically dropped from network queues. To avoid oscillation between intense bursts of traffic and choking off traffic entirely, the rate at which these packets are dropped is ramped up gently after a certain threshold in the queue length is reached. Similarly, in our ED model, we set a minimum threshold, below which ED queue lengths are considered acceptable and no dropping occurs. The rate at which patients are dropped increases linearly with queue length, until some maximum threshold is reached, past which the drop rate remains constant. Since we consider “dropping” (turning away patients) as unacceptable, our model instead considers a drop as a patient redirection to another ED. The mechanism for this is either self-redirection to another ED or redirection by a central dispatch/control.

Two modes were considered: first, where patients are redirected to a random ED with uniform probability, and second, where patients are probabilistically redirected to an ED based on the ratio of doctors to patients waiting. The latter case results in EDs that are less busy assigned a higher likelihood of patients being redirected there. This reflects an assumed patient preference for shorter waiting time, and also demonstrates the utility of city-wide ED status information dissemination. This is contrasted with the former case, where patients are simply guessing as to which ED is a more preferable alternative without external guidance.

To ground our simulations to the greatest extent possible in real data, the work drew on a report on ED usage in Winnipeg, Canada released by the Manitoba Centre for Health Policy [27]. There were 185,659 ED visits in Winnipeg among six hospitals, the breakdown of which by CTAS [28] triage level roughly corresponded to the triage levels used in [20]. While the data on actual treatment times are not readily available to date, the distribution of treatment times were based on triage levels from [20]. Further, no data on the variation in patient arrival rates based on time of day, day of the week, time of year, or variation between individual EDs is available to date, and thus uniform rates are assumed for these variables. However, it should be noted that the simulator readily incorporates these variations and ranges in data, when they become available.

With the information presented, it was possible to estimate arrival rates of patients for each triage level at each simulated ED. An arbitrary but reasonable minimum threshold of 10 patients waiting in the queue was chosen for the RED model. The drop or redirection rate increases linearly to a maximum of 50%, reached at a queue length of 20. It is reasonable to assume that staffing levels at each ED do not match demand. Because arrival rates are uniform among the simulated EDs and to make the simulation interesting, two EDs are staffed with two doctors, two EDs have three doctors, and two EDs have four doctors on staff during the simulation.

As in the staff allocation simulation, three 24-hour scenarios were investigated with ten trials each, and a warm up time of 24 hours. For comparison, the first scenario (labeled No Redirection) assumes that there is no ED status information available and that patients are better off going to the nearest ED and remaining there regardless of queue length and wait times. The second scenario allows redirection based on the RED model, and the destination ED is chosen from a uniform probability. This scenario is labeled Random RED. The third scenario, labeled Guided RED, invokes RED redirection where EDs with lower expected waiting time are probabilistically chosen more often as the destination ED.

**Figure 7:**
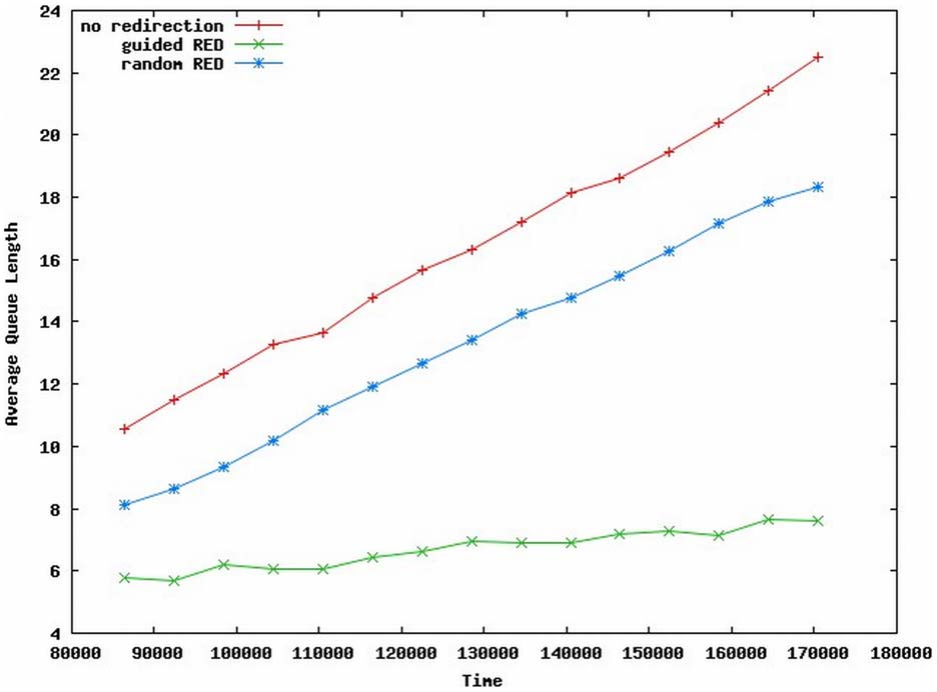
Average Queue Lengths for Various Patient Redirection Policies.

Un-aggregated patient wait times for these scenarios are not shown here, as the variation between individual wait times was very high, likely due to the disparity between ED conditions. As before, results in Figure 7 have a qualitative value, indicating that average queue lengths among all hospitals are shortest for the Guided RED scenario. Also, the overall doctor utilization is highest in the Guided RED scenario. While the model is not currently refined enough to test causal relationships, these two factors appear to be correlated, and it is interesting to note that a significant queue length reduction (waiting time) was achieved with only a modest increase in utilization and no additional resources.

### Detailed ED Queuing Models

The following example is a QM of an individual ED, with potential extensions to a multiple-hospital QM. The example demonstrates the ability of a QM to generate quantitative data that can be used to identify system bottlenecks. While the data are quantitative, the results of this given example should be viewed as qualitative, highlighting the overall relative sensitivities and impacts of changing parameter values. As the model is refined and extended with data of higher accuracy, range, and precision, the results become amenable to statistical analysis for hard metrics, as well as causal and correlational relationships

**Figure 8:**
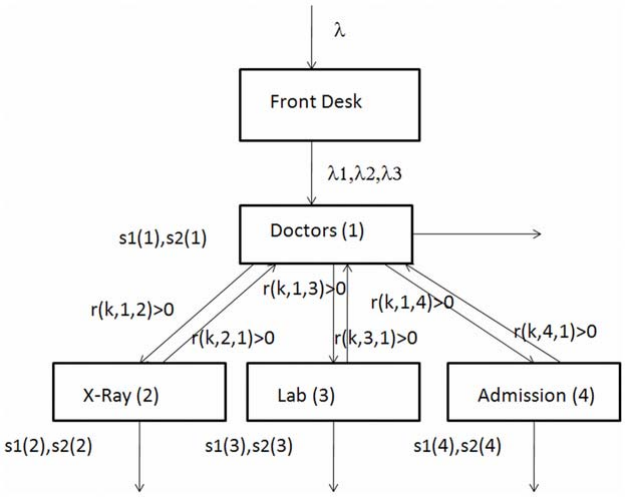
A Four Node Emergency Department Queuing Model.

In the current example, we consider a four-node system illustrated in Figure 8. Node 1 – Doctors, Node 2 – Diagnostic 1, Node 3 – Diagnostic 2, and Node 4 – Admission to facility. Every patient enters the facility and is classified into one of three groups: Class 1 – high priority, Class 2 – next priority and Class 3 – lowest priority. Let s1(n) and s2(n) be the first and second moments of service times at node n for all patients. These parameters represent the average service time and the variance associated with the service. For this illustration, the service times at all nodes are equal for all classes, although there are differences in the order of priority in which patients are attended, as well as differences in how patients move between nodes.

In the example, all patients are assumed to be seen by a healthcare worker able to assess and prescribe treatment specific to the condition (physician, physician-assistant, etc.). At that point, some patients may be discharged, while others are sent to diagnostic services and/or facility admission. Upon completing diagnostics, some patients may again be seen by a physician before discharge. In the QM, let r(k,i,j) be the ratio of class k patients that go from node i, to node j. For example, r(1,1,2) = 0.1 implies that after a class 1 patient finishes seeing the doctor there is 0.1 probability that he/she may be sent to X-Ray.

The example used the following simulated data: patients arrived at the rate of two per hour; 50% of patients are class 1, 25% are class 2, and 25% are class 3. So the arrival rates are 1,0.5,0.5, respectively for the three classes. In Figure 8 these are represented by λ, λ1, λ2, and λ3. Service time data were simulated as follows (in minutes): s1(1) = 20, s1(2) = 5, s1(3) = 10, s1(4) = 60, s2(1) = 500, s2(2) = 30, s2(3) = 200, s2(4) = 8000. We intentionally selected high second moments for admission to allow for high variance. As patients move through the system they are assigned transfer rates. The transfer rates, i.e. probabilities r(k,i,j) are given as: r(k,1,2) = 0.1 for all k, r(k,1,3) = 0.2 for all k, r(1,1,4) = 0.3, r(2,1,4) = 0.2, r(3,1,4) = 0.1; r(1,2,1) = 0.6, r(2,2,1) = 0.5, r(3,2,1) = 0.4; r(1,3,1) = 0.7, r(2,3,1) = 0.2, r(3,3,1) = 0.2; All other r(k,i,j) = 0. These transfer rates enable the modeling of patient flow within the ED.

For this example, we obtain the time spent at a node (waiting and receiving attention) as w(k,n) for class person of Class k at Node n. For the simulated data outlined, the results are shown in Table 1.

**Table 1:** Time spent at nodes as patients of various class flow through the system.

	n = 1	n = 2	n = 3	n = 4
k = 1	27.55	5.03	10.39	94.19
k = 2	57.10	5.09	11.00	171.97
k = 3	121.54	5.13	11.42	228.16

While hypothetical, the numbers illustrate the qualitative differences in system behaviour, as experienced by patients of different priority classes. In the current stage of development, the model provides insight into the relative sensitivities and impacts of varying input parameters, and allows a qualitative feel for varying policies and practices within the ED. Further simulations were carried out to demonstrate this potential. Table 2 illustrates the waiting and service time variations for preemptive and non-preemptive policies.

**Table 2:** Time spent at nodes for preemptive (non-preemptive) for arrivals.

	n = 1	n = 2	n = 3	n = 4
k = 1	27.55 (34.67)	5.03 (5.06)	10.39 (10.76)	94.19 (110.33)
k = 2	57.10 (52.97)	5.09 (5.06)	11.00 (10.80)	171.97 (150.84)
k = 3	121.54 (96.59)	5.13 (5.06)	11.42 (10.84)	228.16 (179.86)

Table 3 illustrates waiting and service times for preemptive and non-preemptive policies, as the arrivals rates are changed to (.25, 1.25, .5).

**Table 3:** Time spent at nodes for preemptive (non-preemptive) for arrivals (.25, 1.25, .5).

	n = 1	n = 2	n = 3	n = 4
k = 1	21.30(29.89)	5.00 (5.05)	10.10 (10.72)	66.17 (89.52)
k = 2	38.28 (41.50)	5.05 (5.05)	10.67 (10.76)	105.17 (105.57)
k = 3	112.21 (88.73)	5.12 (5.06)	11.39 (10.81)	162.76 (130.13)

Table 4 illustrates waiting and service times and for preemptive and non-preemptive policies, as the arrivals rates are changed to (.25, .25, 1.5).

**Table 4:** Time spent at nodes for preemptive (non-preemptive) for arrivals (.25, .25, 1.5).

	n = 1	n = 2	n = 3	n = 4
k = 1	21.30(29.84)	5.01 (5.05)	10.10 (10.72)	66.17 (81.65)
k = 2	25.18 (32.05)	5.03 (5.05)	10.28 (10.73)	77.24 (85.12)
k = 3	62.58 (58.09)	5.08 (5.05)	10.97 (10.78)	102.35 (92.72)

These simple simulations demonstrate the qualitative impacts in waiting and service times of patients, which would be difficult to model otherwise. While based on simulated data, the results imply that the waiting times of each patient class is affected by how they are classified. For example, by placing more patients in the highest priority class, the waiting times increase for *all* patients. These kinds of insights demonstrated by the QM are opportunities for further investigation.

As with ABMs, future efforts will refine and extend the QM model with real data, even though real data may carry a degree of uncertainty to it as well. This encompasses both topological data, a range of patient and staff parameters, and patient flow parameters (for example, service and arrival rates). With an increasingly context-specific data set, considerable studies can be done relative to investigating efficiencies and performance improvement as a function of resources. Going further, comparative modeling of alternative care processes could be carried out as well.

The four-node QM outlined above is extendable to any level of hierarchy or any number of nodes. Furthermore, extending the QMs to encompass multiple EDs and/or alternative care practices is a reasonable extension and does not present significant technical difficulties. As with all the models discussed, the utility of the single- or multi-hospital QM is dependent on accurate topological and flow models with reasonable estimates for all parameters. With increased real data inputs, hard metrics become reasonable model outputs. Initial simple models that rely on simulated data nonetheless provide insight into qualitative relationships between parameters.

### Optimizing Policy: Machine Learning

Optimizing patient access and patient care policies is not necessarily amenable to either deterministic or ad-hoc approaches, as the problems themselves are difficult combinatorial problems. In these cases, significant gains can be made with non-deterministic algorithms guided by analogues to physical systems and/or learning systems, which in turn provide a measure of credibility and confidence in the solution. This final section discusses a genetic programming (GP) technique that mimics evolutionary systems in attempting to optimize towards a solution. The GP approach is one of many possible approaches, but does closely relate to how actual policy and decisions are made in a very difficult problem space.

The ABM development to date for the applications outlined in earlier sections suggested a means to simulate and comparatively assess policy and practices (“what-if” scenarios) prior to implementation. However, this requires a human to generate a policy for the ABM to test. Examples of such policies may be “staff with x number of doctors instead of y number of doctors” or “begin to divert patients once the number of waiting patients exceeds a defined threshold”.

In addition, ABMs can incorporate evolutionary algorithms that allow realistic agent learning, and extensions of this work include the addition of a machine learning (ML) module to the ABM framework, to facilitate automatic policy generation. The model generates policies, uses the ABM to evaluate them, and then uses the best individual policies as the basis for the next generation of policies. This process is iterated until pre-defined criteria are met.

Genetic programming (GP) [29] is one machine learning paradigm for the automatic induction of computer programs through an evolutionary process. The GP paradigm is well established and includes successful research applications in the areas of data mining [30], image classification [31], automatic circuit evolution [32], and robot control [33]. Evolutionary algorithms (EA), a group of algorithms to which GP belongs, can improve upon human generated policies, and sometimes in unexpected ways [34].

Future work will invoke the ABM framework to investigate the viability of using a GP-based machine learning system to forecast ED waiting times and to generate policy. As data collection frameworks such as the one posited in [21] become available, the ML system could be trained and validated on real data. In this instance, the ABM becomes a data generator and an input into the overall ML paradigm.

To develop this extension, refinements to the model parameters are required. One such refinement is to the model of the agent (patient), whereby the patient may change its internal state probabilistically. These internal states may represent, for example, getting less or more ill, leaving the ED, etc, and one can evolve (automatically generate) triage policies to optimize patient flow for these more complex agents. A second refinement may be to generate an architecture-agnostic patient diversion policy, where only the policy is evolved, and the means of implementing the policy is assumed to be in place and is treated as abstract. Third, an agent class responsible for executing patient diversion policies generated by the GP-system will have to be added to the ABM.

**Figure 9:**
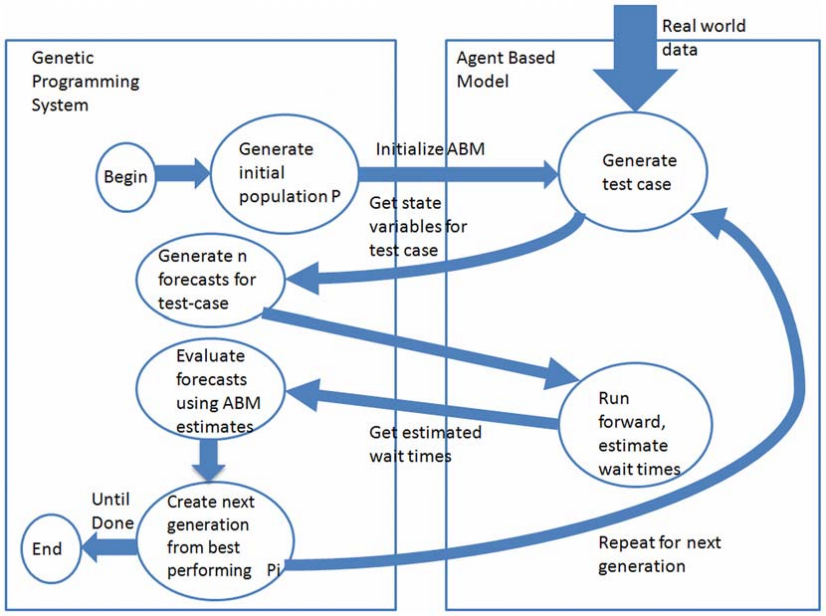
Genetic Programming and Agent Based Model Integration.

To facilitate these extensions, the machine learning paradigm we most closely follow is supervised or reinforcement learning (RL) [35]. Figure 9 illustrates the general nature of how the ABM provides feedback to the GP-based ML system, essentially acting as a data generator until real data can be used to validate, for example, a wait-time forecasting function.

Finally, a further research direction is to utilize ABMs not only for policy shifts within an existing ED, but to develop ABMs for modeling alternative forms of ED care. EDs necessarily function with competing objectives. In many EDs, the objective is to maximize the flow of patients; improvements are derived through staff levels, bed numbers and utilization, hours of operation, diversion policies etc. More recently, there have been aggressive efforts to reconceptualize the ED entirely, around its main function of addressing emergencies (vs. maximizing patient flow) [36]. This has follow-on effects in multiple directions, including but not limited to staff configurations (teams vs. individuals) and the layout of the physical facility. Here ABMs offer a useful tool in guiding decisions around such paradigmatic shifts within an individual institution.

### Summary

This concept paper presented two modeling methodologies applied to investigating patient access and patient waiting times in hospital EDs, within the objective of developing tools that can help guide policy and practice improvements. The first model is an agent based modeling framework, oriented to the simulation of EDs in either stand-alone mode, as multiple interacting EDs, as well as technologies well suited to enhance simulation with statistical empirical data collected in real time. The second model is a more traditional queuing model, whose suitability is discussed for similar applications. Analogues from telecommunications engineering were introduced, selected for their conceptual parallels to the patient access and patient wait time cases, and because the telecommunications analogues have been well vetted within the community, albeit for difference purposes.

The work is developmental, currently relying on coarse-grained approximations, relatively simple general scenarios, and to a large degree on simulated data. At their current stage of development, the models' utility lies in their ability to provide qualitative insights into the relative sensitivities and impacts of model parameters, to illuminate scenarios worthy of more complex investigation, and to iteratively validate the models as they continue to be refined and extended. With an increasing proportion of real data inputs (spatial-topographical as well as agent parameters), accurate and precise system metrics amenable to statistical processing become reasonable model outputs. Both the agent based and the queuing model frameworks are oriented to augmenting simulation with empirical data when available. In this context, the work also presented opportunities in which emerging technologies such as RFID, which carry a high potential as tracking data sources, would significantly enhance the modeling efforts by provisioning the models with context-specific empirical inputs as close to real time as possible. These sources can be mined in a statistically significant manner and provide real world input for the simulation.

The models under development are also open source and rely on open source components. They are extendable and can be ported or tailored to a variety of hospital IT applications, several of which were identified here. Eventually, these tools may be more closely coupled to commercial hospital information systems, thereby providing better optics as to refining and optimizing hospital processes. The final section of the paper provided an overview of future work in augmenting the policies with machine learning, which may be the closest means of simulating decision-making in a complex problem space. Finally, although the emergency department was the main focus of this research, the frameworks discussed are amenable to the study of intra-hospital wards, as well as inter-hospital and hospital-community interactions.

An example of a multiple ED simulation is available on YouTube [37].
